# Oligogenic genetic variation of neurodegenerative disease genes in 980 postmortem human brains

**DOI:** 10.1136/jnnp-2017-317234

**Published:** 2018-01-13

**Authors:** Michael J Keogh, Wei Wei, Juvid Aryaman, Ian Wilson, Kevin Talbot, Martin R Turner, Chris-Anne McKenzie, Claire Troakes, Johannes Attems, Colin Smith, Safa Al Sarraj, Chris M Morris, Olaf Ansorge, Stuart Pickering-Brown, Nick Jones, James W Ironside, Patrick F Chinnery

**Affiliations:** 1 Department of Clinical Neurosciences, University of Cambridge, Cambridge, UK; 2 Department of Mathematics, Imperial College London, London, London, UK; 3 Institute of Genetic Medicine, Newcastle University, Central Parkway, Newcastle upon Tyne, UK; 4 Department of Clinical Neurosciences, John Radcliffe Hospital, Oxford, UK; 5 Newcastle Brain Tissue Resource, Newcastle University, Newcastle upon Tyne, UK; 6 Department of Basic and Clinical Neuroscience, Institute of Psychiatry, Psychology and Neuroscience, King’s College London, Oxford, UK; 7 National CJD Research & Surveillance Unit, Centre for Clinical Brain Sciences, University of Edinburgh, Western General Hospital, Edinburgh, UK; 8 Department of Neuropathology, John Radcliffe Hospital, Oxford, UK; 9 Division of Neuroscience and Experimental Psychology, Faculty of Biology, Medicine and Health, The University of Manchester, Manchester, UK; 10 MRC Mitochondrial Biology Unit, Cambridge Biomedical Campus, Cambridge, UK

**Keywords:** dementia

## Abstract

**Background:**

Several studies suggest that multiple rare genetic variants in genes causing monogenic forms of neurodegenerative disorders interact synergistically to increase disease risk or reduce the age of onset, but these studies have not been validated in large sporadic case series.

**Methods:**

We analysed 980 neuropathologically characterised human brains with Alzheimer’s disease (AD), Parkinson’s disease-dementia with Lewy bodies (PD-DLB), frontotemporal dementia-amyotrophic lateral sclerosis (FTD-ALS) and age-matched controls. Genetic variants were assessed using the American College of Medical Genetics criteria for pathogenicity. Individuals with two or more variants within a relevant disease gene panel were defined as ‘oligogenic’.

**Results:**

The majority of oligogenic variant combinations consisted of a highly penetrant allele or known risk factor in combination with another rare but likely benign allele. The presence of oligogenic variants did not influence the age of onset or disease severity. After controlling for the single known major risk allele, the frequency of oligogenic variants was no different between cases and controls.

**Conclusions:**

A priori, individuals with AD, PD-DLB and FTD-ALS are more likely to harbour a known genetic risk factor, and it is the burden of these variants in combination with rare benign alleles that is likely to be responsible for some oligogenic associations. Controlling for this bias is essential in studies investigating a potential role for oligogenic variation in neurodegenerative diseases.

## Background

Genetic variation in over 50 genes contributes to the risk of developing neurodegenerative diseases.^1–5^ Some of the known risk alleles are common in the general population, raising the possibility that multiple interacting genetic variants might enhance the risk of developing disease or modify the disease phenotype.^6–10^ In keeping with this, some familial cases of frontotemporal dementia-amyotrophic lateral sclerosis (FTD-ALS) appear to have a greater ‘burden’ of variants when compared with controls,^11^ which may explain an earlier age of onset.^8^ However, it is currently not clear whether this also occurs in non-familial FTD-ALS cases or in other neurodegenerative disorders, where previously reported associations could either be due to a single highly penetrant monogenic allele co-associated with benign non-functioning variants, or whether there is a genuine synergistic interaction between two or more functional genetic variants. To address this, we performed comprehensive clinical variant interpretation on exome sequence data and *C9Orf72* genotypes in 980 neuropathologically characterised brains from the MRC Brain Bank Network.

## Methods

We studied the following: Alzheimer’s disease (AD), n=277; FTD-ALS n=244; Parkinson’s disease-dementia with Lewy bodies (PD-DLB), n=97 and neuropathologically normal controls, n=362,^12^ with 97.2% of all individuals studied having no family history of a neurodegenerative disorder (online supplementary table 1). Demographic data including the age of disease onset and death, disease duration and family history of disease, together with the *antemortem* clinical diagnosis and *postmortem* neuropathological diagnosis were available (table 1).

10.1136/jnnp-2017-317234.supp1 Supplementary file 1

**Table 1:** Clinical and demographic data for the major cohorts within the study

Phenotype	Number of cases	Male (number)	Female (number)	Mean age onset (years) (SD)	Mean age death (years) (SD)	Number with FH	Cases with highly penetrant allele or RF	Oligogenic cases (N (%))	Oligogenic cases possessing a penetrant allele or RF (N (%))	Fisher’s test (P value)
Control	362	232 (64.1)	130 (35.9)	N/A	63.3 (18.8)	N/A	N/A			
FTD-ALS	244	143 (58.6)	101 (41.4)	59.4 (11.8)	64.6 (11.7)	14	33	19 (7.78%)	11 (57.9%)	0.0001
AD	277	131 (47.3)	146 (52.7)	65.4 (10.2)	77.7 (11.7)	11	36	6 (2.17%)	6 (100%)	0.0001
DLB	58	36 (62.1)	22 (37.9)	66.7 (8.4)	76.7 (7.0)	2	16	25 (25.78%)	10 (62.5%)	0.0007
PD	39	28 (71.8)	11 (28.2)	59.9 (10.9)	72.3 (9.2)					

Oligogenic was defined by the presence of >1 variant within the relevant disease panel at <1% MAF in the Exome Aggregation Consortium database. Monogenic or cases harbouring genetic risk factors were defined as outlined in the supplementary methods.^11^

AD, Alzheimer’s disease; DLB, dementia with Lewy bodies; FH, family history; FTD-ALS, frontotemporal dementia-amyotrophic lateral sclerosis; MAF, Minor allele frequency; N/A, not available; PD, Parkinson’s disease.

Exome sequencing was restricted to on-target homozygous, heterozygous and compound heterozygous variants with a minimum read depth of 10, and base quality score of 20 across the 980 subjects, where the variant allele frequency (VAF) was <5% in the Exome Aggregation Consortium (ExAC).^13^ Ingenuity Variant Analysis was used to study 49 genes known to be associated with neurodegenerative disorders (see online supplementary table 2). The 49 genes were subsequently grouped into six gene panels: AD panel (n=8), PD-DLB panel (n=16), full FTD-ALS panel (n=28), medium FTD-ALS panel based on that previously described^3^ (n=12) and a small FTD-ALS panel as previously described^11^ (n=5), together with the entire panel (n=49 genes). All panels were filtered for variants present at VAF <1% and <5% *C9orf72* genotypes^12^ as stated.

Pathogenic (P) or likely pathogenic (LP) variants were defined using the American College of Medical Genetics (ACMG) criteria^14^ as described,^12^ together with known genetic risk factors. Other variants identified as benign (B), likely benign (LB) or of uncertain significance (US) based on the ACMG criteria, and the remaining variants (VAF 0.5%–5% in monogenic genes, or non-risk factor variants in risk-factor genes) were annotated as unclassified (UC). Oligogenic individuals were defined as those who had two or more non-synonymous, frameshift or stop-loss or gain-inducing point mutations in the relevant panel (as stated), or those who tested positive for the *C9orf72* hexanucleotide repeat expansion plus had at least one of the point mutation within the panel.

## Results

Across the entire cohort of 980 subjects, we observed a total of 57 genetic variants in the AD gene panel, 141 variants in the primary FTD-ALS gene panel and 140 in the PD-DLB gene panel (table 1). Six AD cases (2.17%) had >1 variant in the AD panel, and 19 cases (7.79%) of primary FTD-ALS had >1 variant in the primary FTD-ALS panel. These proportions were no different to control subjects (control subjects for the AD panel: 5/362, 1.38%, P=0.545; and full FTD-ALS panel: 26/362, 7.18%, P=0.14). In contrast, 23 cases of PD-DLB (23.71%) had >1 variant in PD-DLB genes, which was greater than controls (controls: 37/362, 10.22%, P=0.004, see online supplementary table 3).

Based on ACMG criteria for pathogenicity,^12^ only three individuals in the entire study (0.30% of n=980) harboured >1 pathogenic, likely pathogenic or known risk factor for a neurodegenerative disease. One patient with DLB (0.1% of n=980) (age of onset in 60s, and death early 70s) had a *LRRK2* p.M1646T mutation associated with PD, and a *TREM2* p.R62H mutation associated with AD.^15^ A patient with AD developing in the seventh decade of life had a *PSEN2* p.L204I mutation and the *TREM2* p.R62H risk factor. A third patient who had early onset PD (onset age fourth decade) due to a compound heterozygous mutation in *PARK2* (p.G430D/pR275W) also had the p.R98W *TREM2* possible risk factor for AD,^16^ but displayed no evidence of any amyloid deposition at *postmortem* (see online supplementary table 8,9).

**Figure 1:**
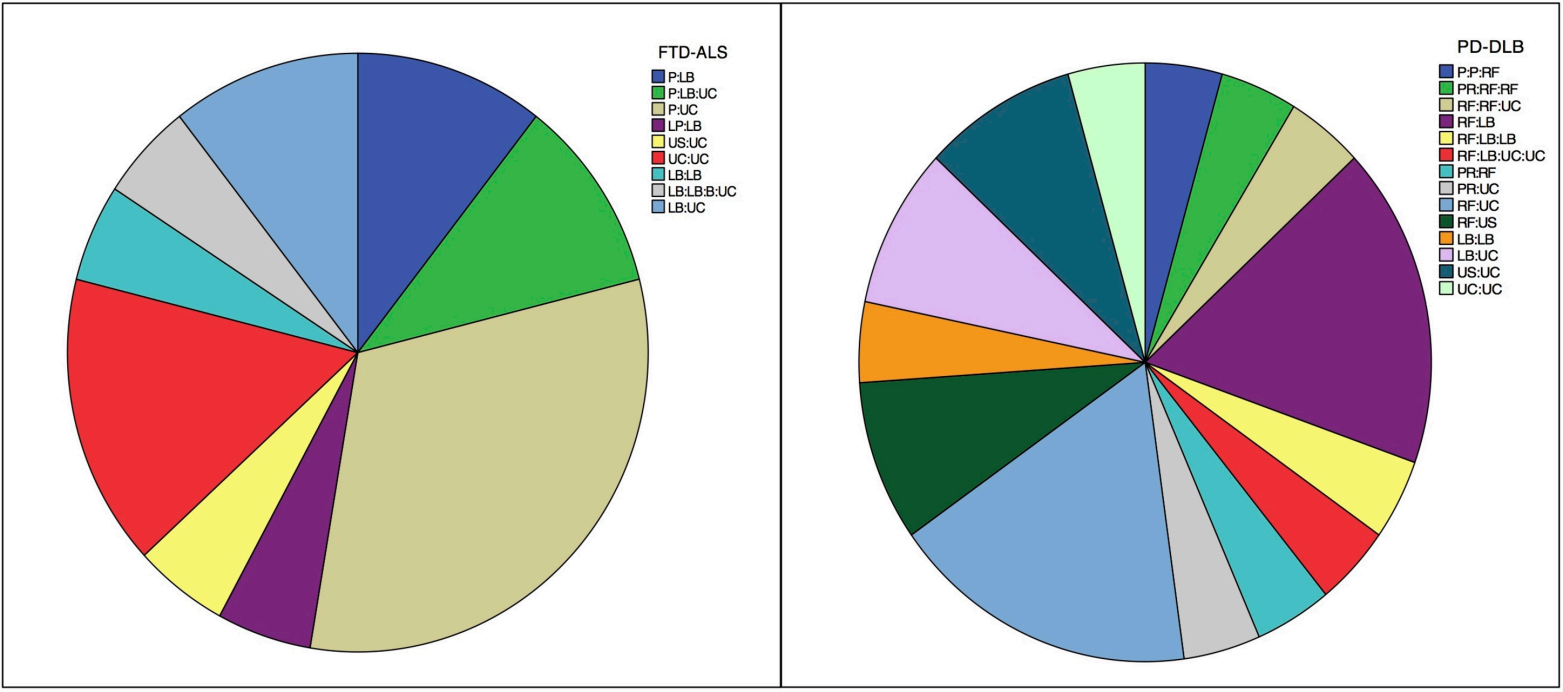
American College of Medical Genetics (ACMG) variant criteria for all cases within the frontotemporal dementia-amyotrophic lateral sclerosis (FTD-ALS) and Parkinson’s disease-dementia with Lewy bodies (PD-DLB) cohorts that had >1 variant within their respective disease panels at 1% MAF. The relative combination of alleles can be seen in the top right of each cohort’s chart. Key references to ACMG classification of each variant: P, pathogenic; LP, likely pathogenic; RF, risk factor; PR, pathogenic in the recessive state (but considered likely benign in the heterozygous state); LB, likely benign; US, uncertain significance; UC, uncategorised.

We observed a significant enrichment of highly penetrant alleles or risk factors within ‘oligogenic’ cases in all disease cohorts (see online supplementary table 4). In FTD-ALS, 11 of the 19 oligogenic cases (57.9%) contained one highly penetrant allele or risk factor within the primary panel, giving the presence of oligogenic variation a positive predictive value (PPV) to identify an individual as someone carrying a pathogenic mutation or known risk factor at 57.9% (95% CI 33.5% to 79.8%) (see online supplementary table 5). We subsequently varied the panel size to reflect published approaches,^3 11^ raised the MAF to 5% within each panel and removed *C9orf72* data from the analysis. In all of these permutations, there was a significant over-representation of highly penetrant allele or risk factor carriers within the oligogenic cohort (figure 1, see online supplementary table 4,5). The same enrichment for highly penetrant alleles within ‘oligogenic’ cases was seen in the AD panel at 1% (PPV 100%, 95% CI 54.1% to 100.0%) and PD-DLB panel (PPV 43.5%, 95% CI 23.2% to 65.5%) (see online supplementary table 5). We subsequently combined all data to employ Bayesian mathematical modelling (see online supplementary data file 2), which showed that the presence of >1 variant within a relevant disease gene panel in an affected individual confers an 80% posterior predictive probability that they have a monogenic allele or risk factor for that disease.

10.1136/jnnp-2017-317234.supp2 Supplementary file 2

We then investigated whether the enrichment of monogenic alleles or risk factors within oligogenic cases was due to a greater overall background mutation rate in these individuals as previously suggested in some genotypes of PD,^17^ but found no evidence of such an association (see online supplementary table 6).

Finally, we removed all cases possessing a highly penetrant allele or risk factor, and compared remaining oligogenic cases of PD-DLB and FTD-ALS with controls (n=362). Based on this analysis, there was no difference in either the proportion of ‘oligogenic’ cases or the mean pathogenicity defined by both SIFT or Polyphen2 score (see online supplementary table 7-9, figures 1−6) between any study group. We also observed no difference in the age of onset, age of death or disease duration between remaining oligogenic cases compared with those with <2 variants (see online supplementary figures 7,8), including the *C9orf72* expansion in the presence of additional variants (see online supplementary figure 9).

## Discussion

With ever more comprehensive panels of genetic testing in neurodegenerative disorders, the possibility of detecting more than one rare variant in an individual will become increasingly likely, posing significant diagnostic challenges and difficulties for genetic counselling. Our data show the observed frequency of ‘oligogenic’ variation is linked to the size of the gene panel and MAF threshold, ranging from 1.4% (AD panel) to 13.3% (PD-DLB panel) in both affected and unaffected individuals (see online supplementary table 3). This highlights that, while each allele is in itself rare, it is not uncommon for any individual to have more than one rare variant across a small disease gene panel. This should be borne in mind when investigating the possibility of an oligogenic mechanism, particularly given the increasing number of genes identified as causing or contributing to neurodegenerative disorders.

Why are our conclusions different to previous studies that were of a similar size? In order to be defined as ‘oligogenic’, an individual must have >1 variant in a known relevant risk gene. This introduces a systematic bias, whereby affected individuals are more likely to harbour one of these alleles than healthy aged individuals. This has been seen previously,^8^ where 10/18 (55.6%) of the ‘oligogenic’ cases had an established highly penetrant allele already known to cause an earlier onset disease (see online supplementary data file 1). The presence of these known risk alleles, in conjunction with a background rate of polymorphic variation, inevitably results in individuals with a known highly penetrant allele or risk factor being more likely to fall into the ‘oligogenic’ group. In keeping with this, our analysis shows that the vast majority of individuals defined as having ‘oligogenic’ variation do indeed have a known risk allele or highly penetrant variant, explaining the initial association we observed between oligogenic variants and PD-DLB. Importantly, after excluding the known major variant in individual cases there was no association between the benign oligogenic variation and neurodegenerative disease or the age of onset.

This same systematic bias will lead to the apparent enrichment of ‘oligogenic’ variants in familial cases. By being familial, these individuals are more likely to harbour a known risk genetic factor, which when combined with the background variant carrier rate, makes them more likely to be classified as oligogenic than healthy controls. Thus, a priori, being a familial case will make it more likely for an individual to have oligogenic variants. This does not necessarily mean that the additional variants are having an effect on the risk of being a familial case. Given the frequency of any individual harbouring two or more variants, and the likely diminishing impact of each variant on the phenotype and disease risk, substantially larger datasets (eg, n>10 000) will be required to definitively resolve this complex issue with robust variant pathogenicity interpretation.
