# Supplementary material for: Oligogenic genetic variation of neurodegenerative disease genes in 980 postmortem human brains
Source: J Neurol Neurosurg Psychiatry. 2018 Jan 13;89(8):813–6. doi: 10.1136/jnnp-2017-317234 (PMC6204946; doi:10.1136/jnnp-2017-317234)
Supplement: Supplementary file 1 [file jnnp-2017-317234supp001.pdf]

SUPPLEMENTARY FILE

SUPPLEMENTARY METHODS

Clinical stratification of cases: Demographic data (age of disease onset and death, disease duration and family history of disease), together with the *ante mortem* clinical diagnosis and *post mortem* neuropathological diagnosis were recorded for all cases. Quantitative neuropathological scores and stages (e.g. Braak neurofibrillary tangle stage [1]) were also supplied for each case as appropriate. Given the known neuropathological and genetic overlap, cases with either frontotemporal dementia (FTD), motor neuron disease (MND)/amyotrophic lateral sclerosis (ALS), were included together in an FTD-ALS group .

Molecular genetic and bioinformatic analysis: DNA extraction, exome sequencing and variant interpretation were performed as previously described[2]. Briefly, DNA was extracted from either the cerebellum, cerebral cortex or basal ganglia. Automated DNA extraction was performed using a DNA extraction robot (Qiasymphony SP robot; Qiagen, Hilden, Germany). Tissue was lysed in 180 µl of ATL buffer (Qiagen, Hilden, Germany) and 20 µl of Proteinase K (Qiagen, Hilden, Germany). Lysates were incubated overnight at 56 °C and at 900 rpm before being loaded onto the Qiasymphony robot. Subsequent extraction was performed using the Qiasymphony DNA mini kit reagents (Qiagen, Hilden, Germany), as per manufacturers protocol. DNA yield was measured using the Nanodrop-8000 Spectrophotometer (NanoDrop Technologies).

Exome sequencing and analysis: Genomic DNA was fragmented, exome enriched and sequenced (Nextera Rapid Exome Capture 62Mb and HiSeq 2000, 100 bp paired-end reads). Bioinformatic analysis was performed using an in-house pipeline including alignment (human reference genome hg19, UCSC) using Burrows-Wheeler Aligner (BWA)[3]. Variant calling was performed using FreeBayes [4]. Subsequent analysis was restricted to on-target homozygous, heterozygous, and compound heterozygous variants with a minimum read depth of 10 in any case or control, and base quality score of 20 within the cohort. Further analysis was performed on frameshift, in-frame indel, or start/stop codon change, missense variants, and splice site loss variants with a minor allele frequency <1% or <5% in the 1000 Genome Project Database[5], European American cases from the NHLBI ESP exomes database[6 7], and ExAC server[8 9], using Qiagen Ingenuity Variant Analysis software.

Variant interpretation: Variants in genes known to cause familial forms of neurodegenerative disease with the appropriate inheritance pattern were previously assessed in all cases according to both the 2015 American College of Medical Genetics (ACMG) [10], and the MacArthur Criteria [11], irrespective of phenotype or neuropathological diagnosis. The ACMG criteria are the primary established criteria for the interpretation of sequencing variants in a clinical context, and the MacArthur criteria are a second stringent criteria that propose that researchers summarise and present a spectrum of evidence in order to truly attribute pathogenicity of identified alleles in sequencing studies.

Ante mortem clinical and *post mortem* pathological data were reviewed for all cases, together with the collation of the MacArthur criteria. Variants were

then classified as either Pathogenic, Likely Pathogenic, of Uncertain Significance, Likely to be benign, or Benign according to the ACMG criteria based on this data and their presence in an appropriate phenotype and with appropriate corresponding pathology. Importantly, each allele was assessed at the allele level, and no consideration was taken as to whether a previous pathogenic allele or risk factor had been identified within that individual.

Defining 'oligogenic' individuals; Individuals with >1 non-synonymous variant (either heterozygous or homozygous) within a their respective panel of genes were classified as 'oligogenic'. This included heterozygous variants in genes that cause monogenic forms of disease in the heterozygous state and those genes that act as known risk factors.

Discussion of oligogenic cases detected by Cady et al[12].

Cady et al[12] identified 18 cases which had oligogenic variation in their study in 2015. By the authors own criteria two of these cases were likely to be homozygous recessive mutations; *SOD1* p.D91A and *SETX* p.I2547T, and two potential compound heterozygous cases; *SETX* p.C1554G and p.R168Q, and *SETX* p.I2547T and p.T14I were also present. They also had 3 individuals with the pathogenic *C9Orf72* hexanucleotide repeat expansion[13], one individual with the pathogenic heterozygous *FUS* p.P525L mutation[14 15], and one with the pathogenic heterozygous *SOD1* p.G38R mutation[16]. Taken together, we therefore suggest that at least 10 of their putative 18 oligogenic cases therefore have genetically determined forms of disease caused by a single allele rather than though a synergistic effect. Without knowing the nature of the rest of their cohort, given that the vast majority of cases were clinically sporadic (89.3%) it is

highly likely that the 55.6% of monogenic cases within the oligogenic cohort was significantly in excess of that observed in the non-oligogenic cohort.

SUPPLEMENTARY FIGURES

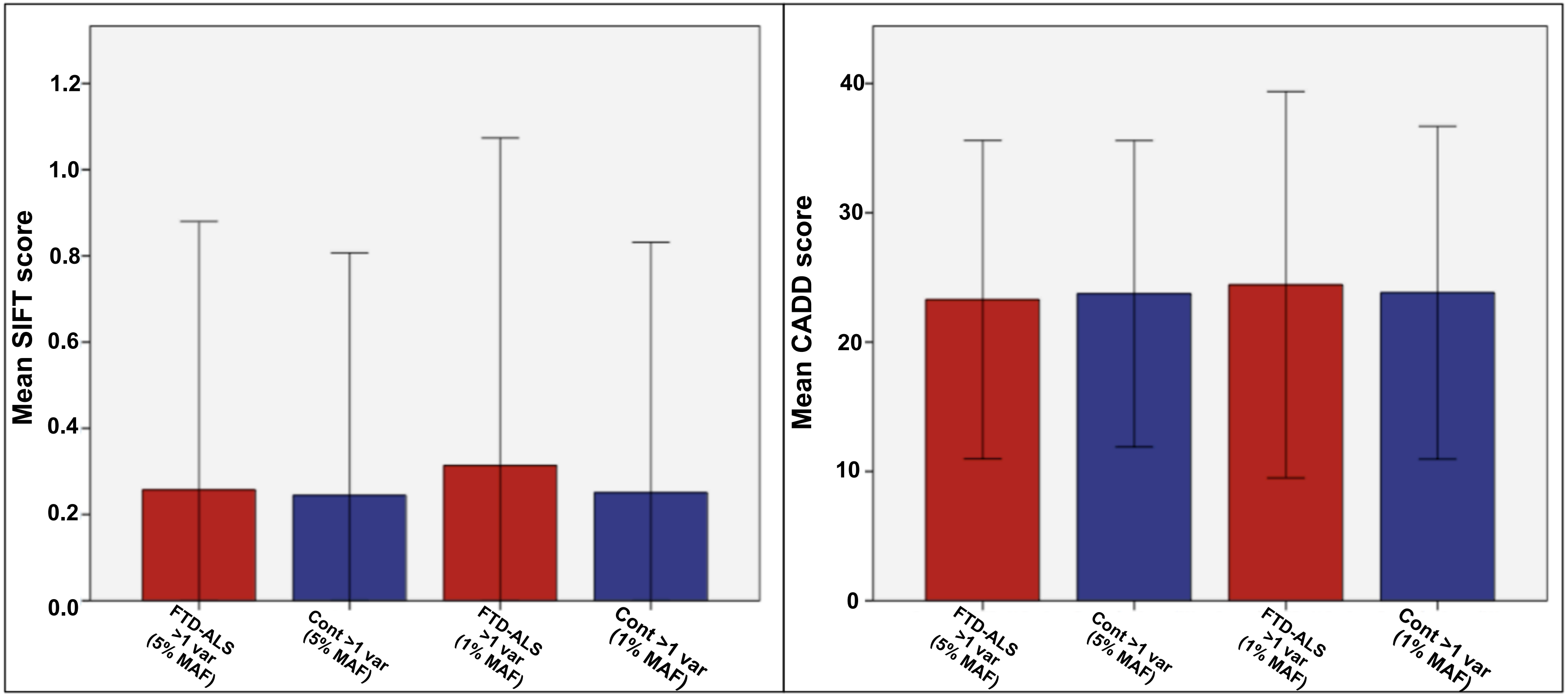

Supplementary figure 1. Mean SIFT and CADD *in-silico* pathogenicity scores for variants in FTD-ALS cases (n=X) and controls (n=362) who have >1 variant, and of which neither variant was deemed to be either a pathogenic, or likely pathogenic variant based on ACMG criteria, nor an established risk factor for disease. There were no significant differences between cases or controls in either criteria at the 5% MAF or 1% MAF threshold (p>0.05, un-paired t-test). Error bars indicate standard deviation from mean.

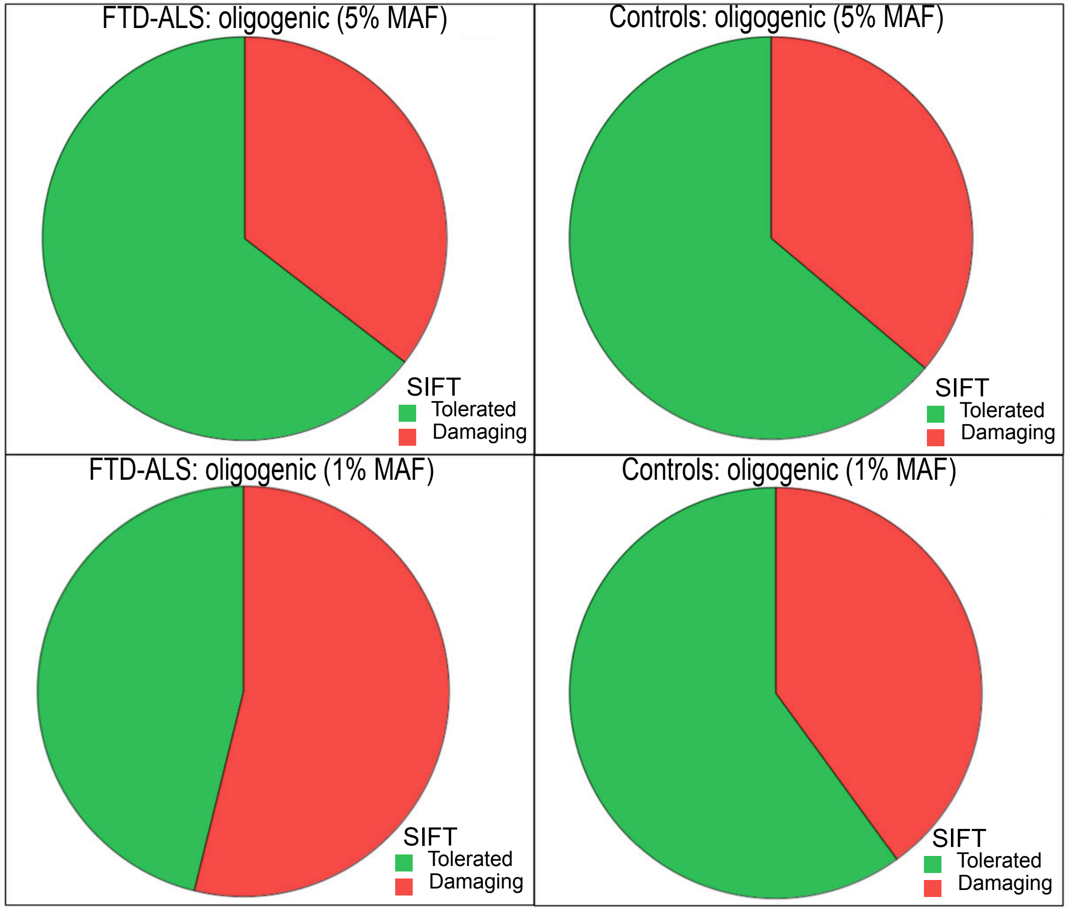

Supplementary figure 2. The proportion of predicted damaging and tolerant SIFT *in-silico* pathogenicity scores for variants in FTD-ALS cases (n=211) and controls (n=362) who have >1 variant, and of which neither variant was deemed to be either a pathogenic, or likely pathogenic variant based on ACMG criteria, nor an established risk factor for disease. There were no significant differences between cases or controls in either criteria at the 5% MAF or 1% MAF threshold (P.0.05, Fisher's exact test).

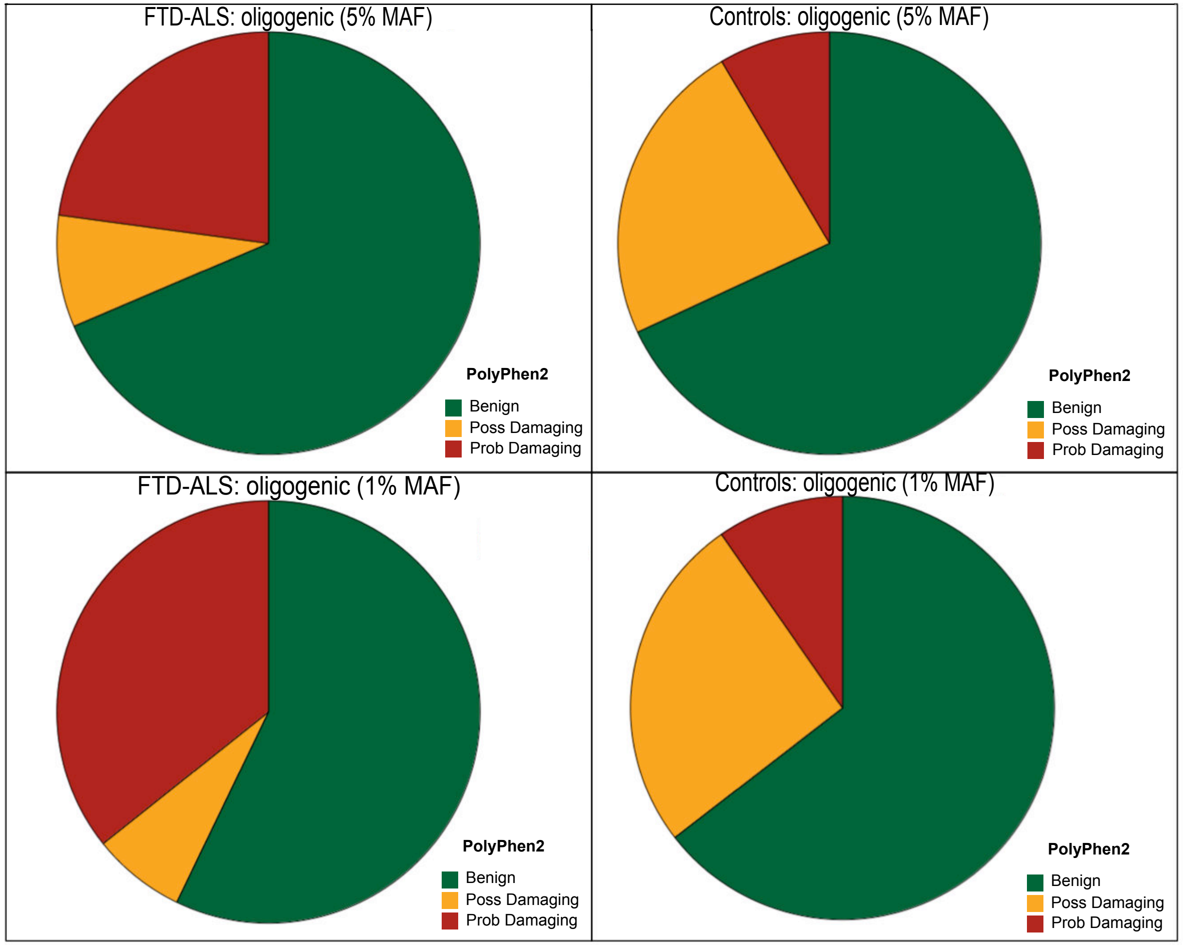

Supplementary figure 3. The proportion of predicted benign, possibly damaging or damaging with PolyPhen2 for variants in FTD-ALS cases (n=211) and controls (n=362) who have >1 variant, and of which neither variant was deemed to be either a pathogenic, or likely pathogenic variant based on ACMG criteria, nor an established risk factor for disease. There were no significant differences between cases or controls in either criteria at the 5% MAF or 1% MAF threshold (P.0.05, Fisher's exact test).

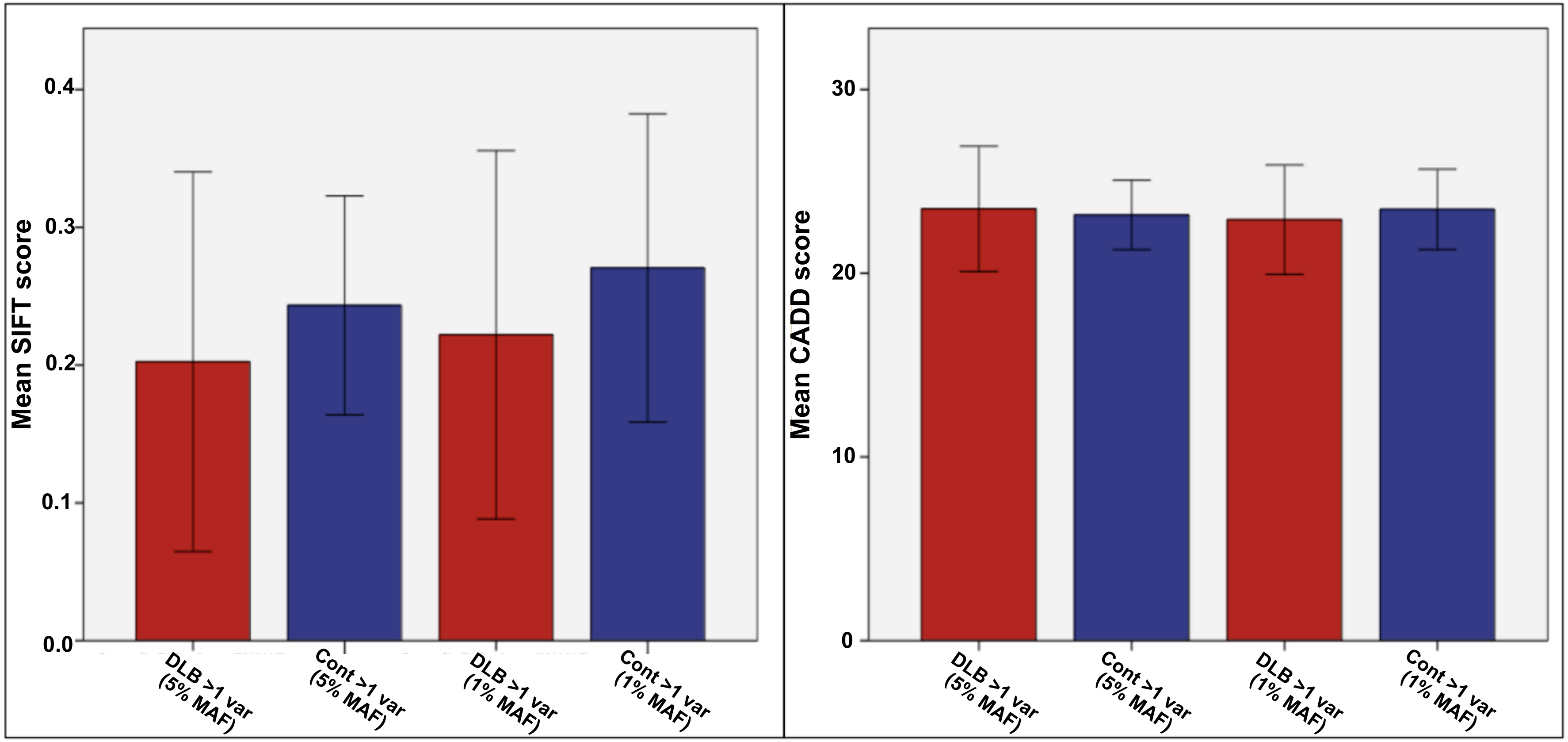

Supplementary figure 4. Mean SIFT and CADD *in-silico* pathogenicity scores for variants in PD-DLB cases (n=97) and controls (n=362) who have >1 variant, and of which neither variant was deemed to be either a pathogenic, or likely pathogenic variant based on ACMG criteria, nor an established risk factor for disease. There were no significant differences between cases or controls in either criteria at the 5% MAF or 1% MAF threshold (p>0.05, un-paired t-test). Error bars indicate standard deviation from mean.

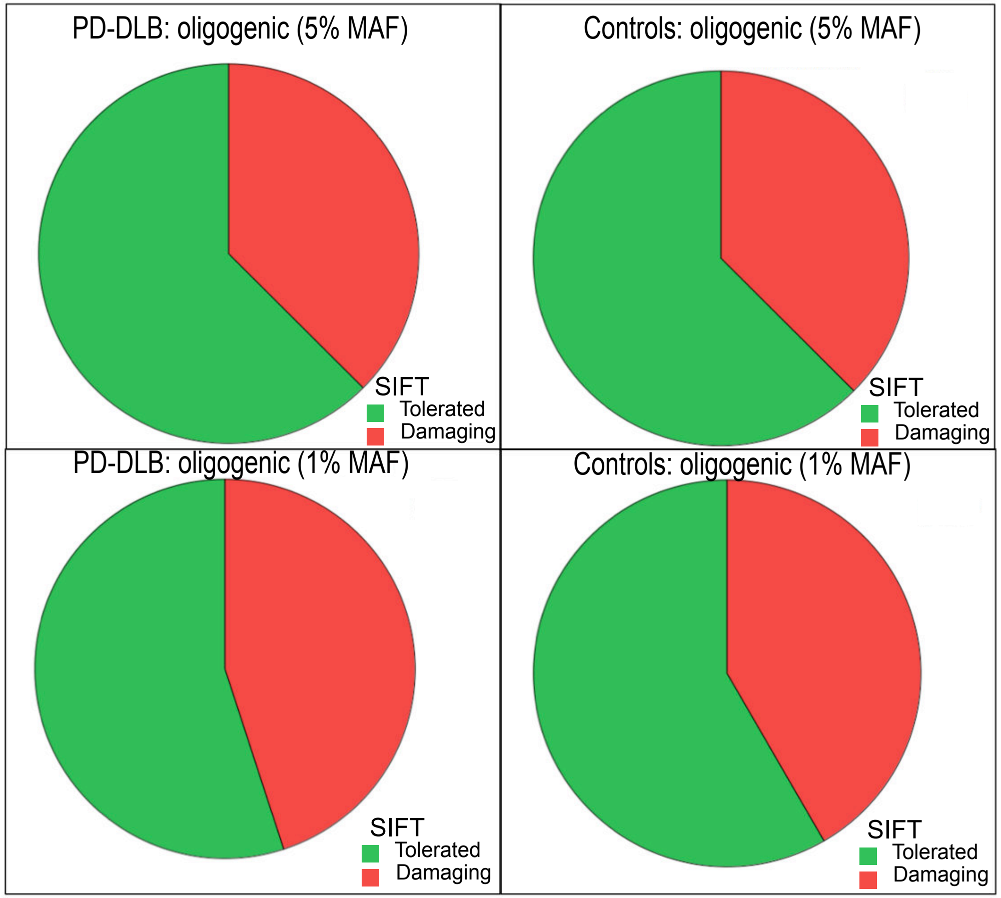

Supplementary figure 5. The proportion of predicted damaging and tolerant SIFT *in-silico* pathogenicity scores for variants in PD-DLB (n=97) and controls (n=362) who have >1 variant, and of which neither variant was deemed to be either a pathogenic, or likely pathogenic variant based on ACMG criteria, nor an established risk factor for disease. There were no significant differences between cases or controls in either criteria at the 5% MAF or 1% MAF threshold (P.0.05, Fisher's exact test).

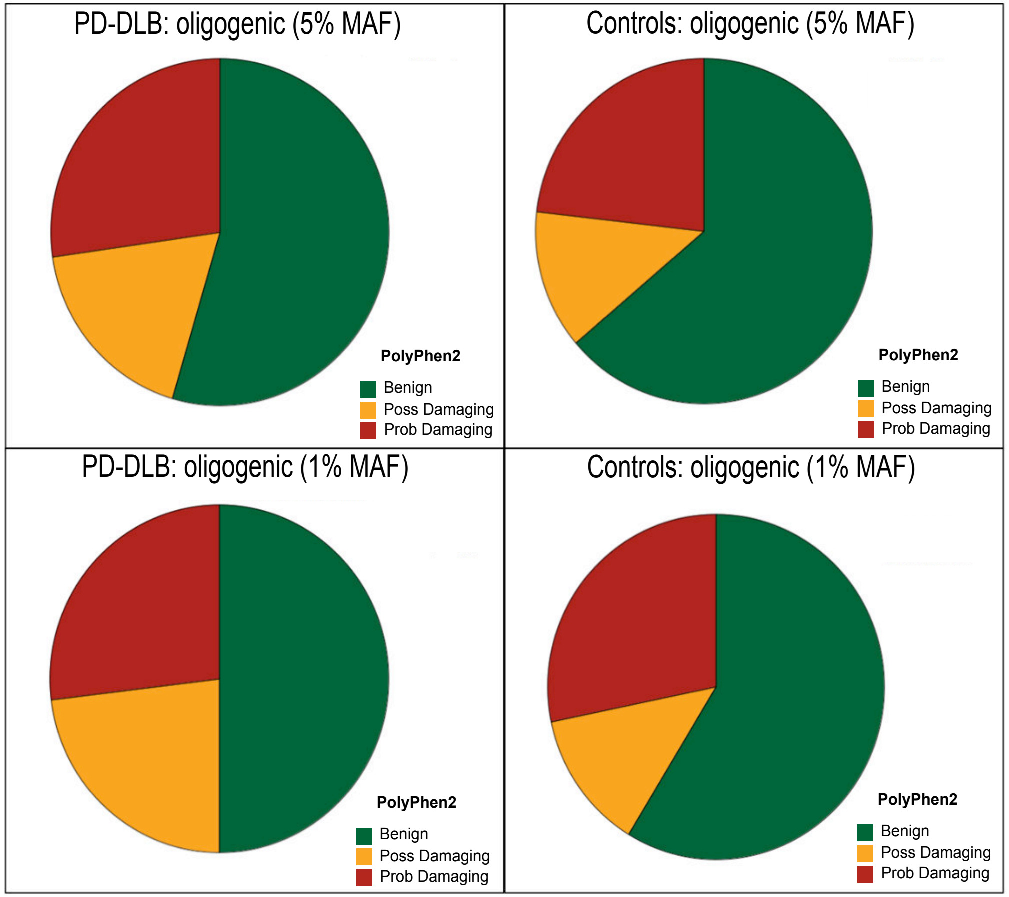

Supplementary figure 6. The proportion of predicted benign, possibly damaging or damaging with PolyPhen2 for variants in PD-DLB cases (n=97) and controls (n=362) who have >1 variant, and of which neither variant was deemed to be either a pathogenic, or likely pathogenic variant based on ACMG criteria, nor an established risk factor for disease. There were no significant differences between cases or controls in either criteria at the 5% MAF or 1% MAF threshold (P.0.05, Fisher's exact test).

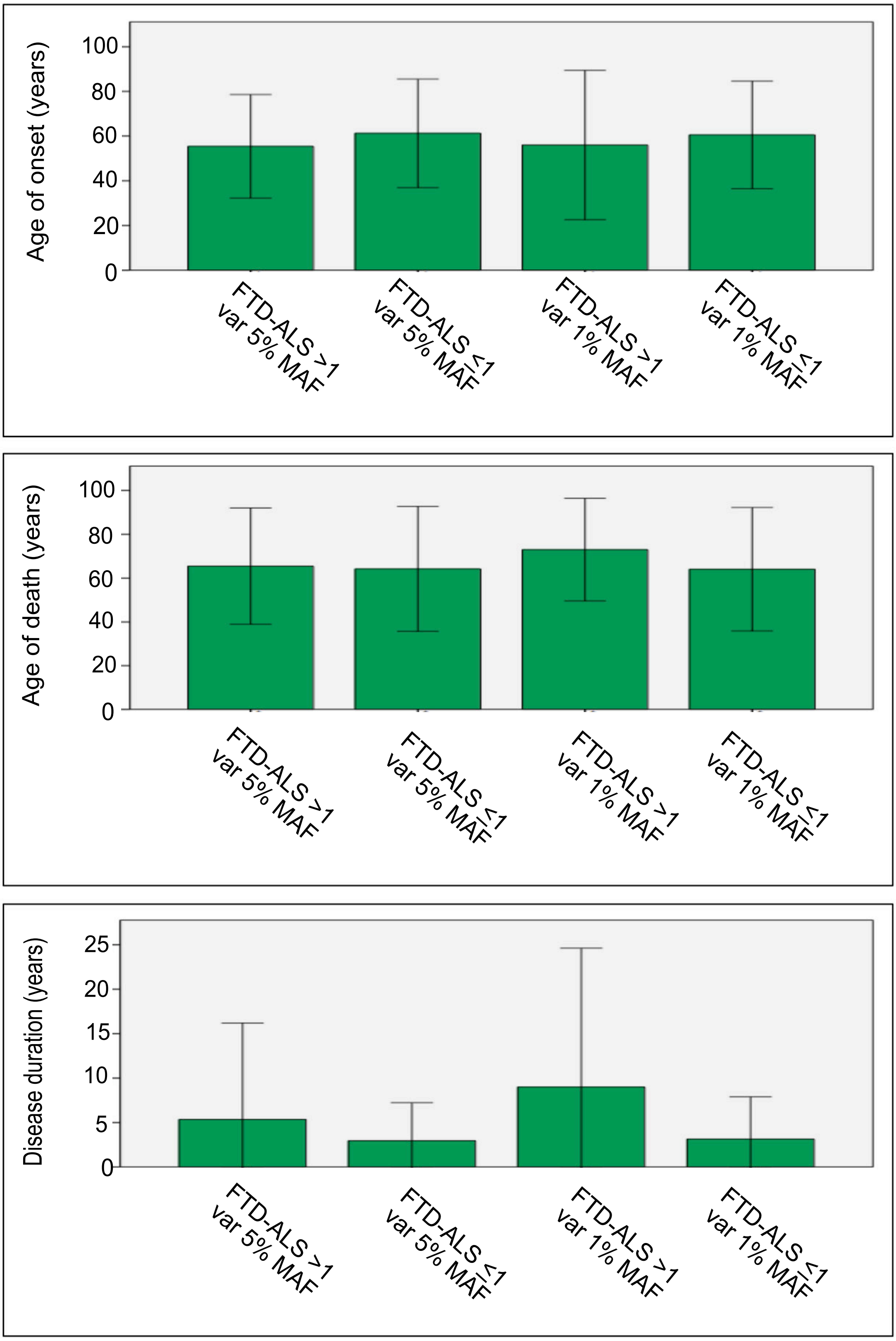

Supplementary figure 7. Mean age of disease onset (top panel), death (middle panel) and mean disease duration (bottom panel) for all cases that have either ≥2 variants in the FTD-ALS gene panel (n=28) at the defined MAF threshold, compared to those with ≤1 variant. There were no differences between cohorts for any criteria. Error bars indicate standard deviation from mean.

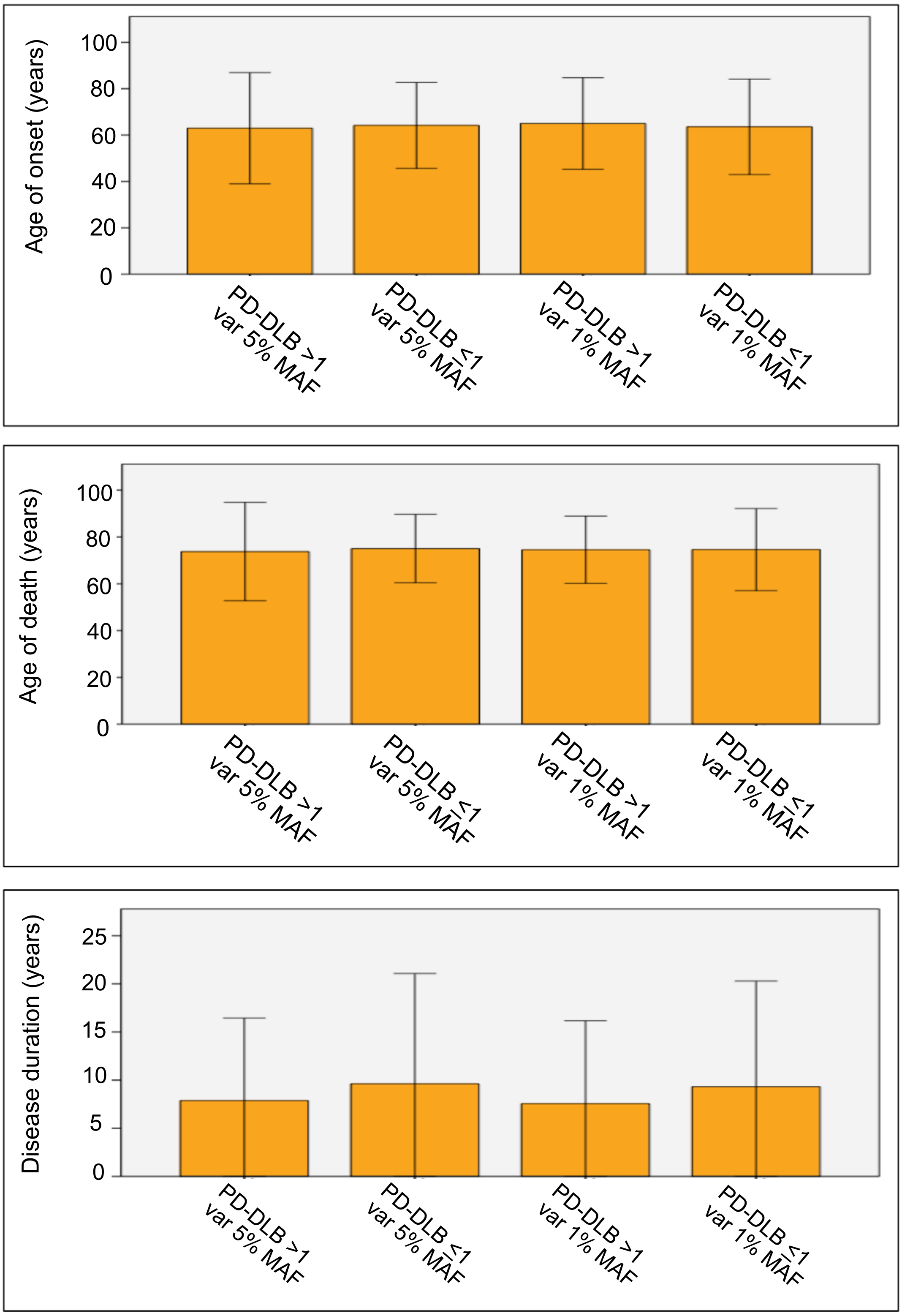

Supplementary figure 8. The mean age of disease onset (top panel), death (middle panel) and mean disease duration (bottom panel) for all cases that have either ≥2 variants in the PD-DLB gene panel (n=20) at the defined MAF threshold, compared to

those with ≤1 variant. There were no differences between cohorts for any criteria. Error bars indicate standard deviation from mean.

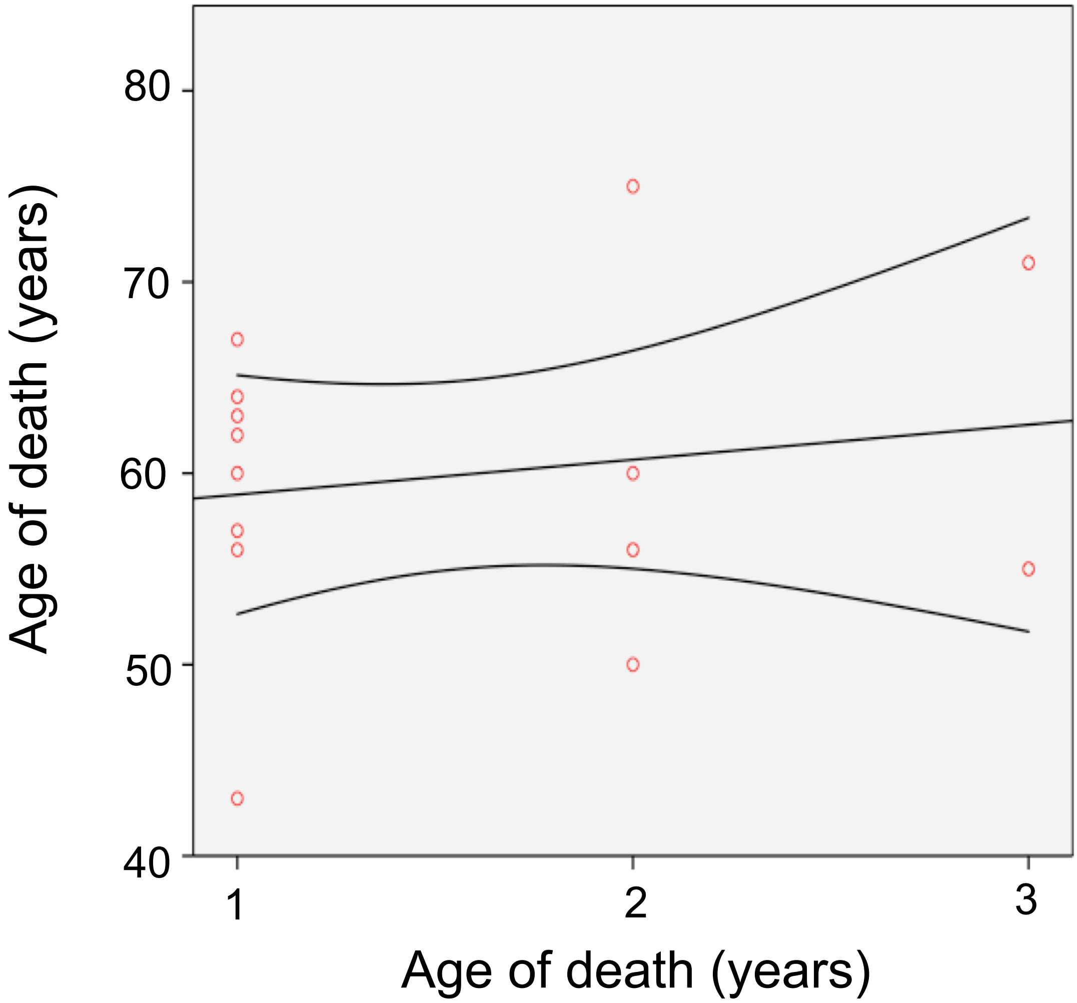

Supplementary figure 9. The mean age of death for all cases (n=14) that carried the C9orf72 mutation against the number of additional non-synonymous variants they possessed within the full FTD-ALS panel at 1% MAF. The line of best fit together with 95% CI is shown. There was no association between the age of death and the number of variants (r^2 = 0.0064).

SUPPLEMENTARY TABLES

Phenotype	Number of cases	Male (number)	Female (number)	Mean age onset	Mean age death	Number with FH
**Control**	362	232 (64.1)	130 (35.9)	N/A	63.3 (18.8)	N/A
**AD**	277	131 (47.3)	146 (52.7)	65.4 (10.2)	77.7 (11.7)	11
**FTD-ALS**	244	143 (58.6)	101 (41.4)	59.4 (11.8)	64.6 (11.7)	14
**DLB**	58	36 (62.1)	22 (37.9)	66.7 (8.4)	76.7 (7.0)	2
**PD**	39	28 (71.8)	11 (28.2)	59.9 (10.9)	72.3 (9.2)	0

Supplementary table 1. Clinical and demographic data for all disease cohorts.

Gene	Disease	Inheritance	Full panel	AD panel	PD-DLB panel	Full FTD-ALS panel (n=28 genes)	Medium FTD-ALS panel (n=12 genes)	Small FTD-ALS panel (n=5 genes)
SNCA	PD	AD/RF	Y		Y			
PARK2	PD	AR	Y		Y			
PINK1	PD	AR	Y		Y			
EIF4G1	PD	AD	Y		Y			
GIGYF2	PD	AD/RF	Y		Y			
HTRA2	PD/AD	AD	Y	Y	Y			
UCHL	PD	AD	Y		Y			
SPG11	PD	AR	Y		Y			
VPS35	PD	AD	Y		Y			
FBX07	PD	AR	Y		Y			
APP	AD	AD	Y	Y	Y			
PSEN1	AD	AD	Y	Y	Y			
PSEN2	AD	AD	Y	Y	Y			
c9orf72	FTD / ALS	AD	Y			Y		Y
GRN	FTD/AD	AD	Y	Y	Y	Y		
CHCHD10	FTD	AD	Y			Y		
TARDBP	FTD	AD	Y			Y	Y	Y
SOD1	ALS	AD/AR	Y			Y	Y	Y
FUS	ALS	AD	Y			Y	Y	Y
PFN1	ALS	AD	Y			Y	Y	
hnRNPA2B1	ALS	AD	Y			Y		
hnRNPA1	ALS	AD	Y			Y		
SETX	ALS	AR	Y			Y	Y	
VAPB	ALS	AD	Y			Y	Y	
OPTN	ALS	AR	Y			Y	Y	
VCP	ALS	AD	Y			Y	Y	
DAO	ALS	AD	Y			Y	Y	
ANG	ALS	AD	Y			Y	Y	Y
DCTN1	ALS	AD	Y			Y	Y	
PARK7	PD	AR	Y					
CHMP2B	FTD/ALS	AD	Y			Y		
SQSTM1	FTD/ALS	AD/RF	Y			Y	Y	
PRPH	ALS	AR	Y			Y		
DPP6	ALS	AR	Y			Y		
MATR3	ALS	AD	Y			Y		
MAPT	FTD/AD	AD	Y	Y	Y	Y		
ALS2	ALS	AR	Y			Y		
SIGMAR1	ALS	AD	Y			Y		
UBQLN2	FTD	XLD	Y			Y		
NOTCH3	CADASIL	AD	Y					
PRNP	fCJD	AD	Y					
COQ2	MSA	AD/AR	Y			Y		
GBA	DLB	RF	Y		Y			
LRRK2	PD/DLB	RF	Y		Y			
TREM2	AD	RF	Y	Y	Y			
SCARB2	DLB	RF	Y		Y			
PON1	ALS	RF	Y			Y		
PON3	ALS	RF	Y			Y		
APOE	AD	RF	Y	Y	Y			

Supplementary table 2. A table of all genes included in each panel in the study, and as indicated in the text.

Panel	Total disease cohort	Disease cases with >1 variant	% of cases >1 variant	Control cohort size	Control cases with >1 variant	Percentage of controls with >1 variant	Fisher's test (cases with >1 variant vs controls)	Fisher's test (cases with >1 variant vs controls) after monogenic or RG cases removed
**>1 variant: full FTD-ALS panel (MAF 5%)**	244	48	19.67	362	48	13.26	**0.04**	0.45
**>1 variant: full FTD-ALS panel (MAF 5%)**	244	43	17.62	362	48	13.26	0.164	0.45
**>1 variant: full FTD-ALS panel (MAF 1%)**	244	19	7.79	362	26	7.18	0.875	0.14
**>1 variant: full FTD-ALS panel (MAF 1%)**	244	15	6.15	362	26	7.18	0.742	0.14
**>1 variant: medium FTD-ALS panel (MAF 5%)**	244	15	6.15	362	15	4.14	0.258	0.50
**>1 variant: medium FTD-ALS panel (MAF 5%)**	244	11	4.51	362	15	4.14	0.839	0.82
**>1 variant: medium FTD-ALS panel (MAF 1%)**	244	7	2.87	362	8	2.21	0.61	0.34
**>1 variant: medium FTD-ALS panel (MAF 1%)**	244	4	1.64	362	8	2.21	0.77	0.34
**>1 variant: AD panel (MAF 5%)**	277	8	2.89	362	10	2.76	1	0.057
**>1 variant: AD panel (MAF 1%)**	277	6	2.17	362	8	2.21	1	0.16
**>1 variant: PD-DLB panel (MAF 5%)**	97	39	40.21	362	92	25.41	**0.0002**	0.70
**>1 variant: PD-DLB panel (MAF 1%)**	97	23	23.71	362	37	10.22	**0.0011**	0.363

Supplementary table 3. A table of the number and frequency of cases with >1 variant in each cohort and in each panel in the study. The proportion of cases with >1 variant in cases compared to controls were first tested before re-testing was performed after the removal of cases that harbour pathogenic variants, likely pathogenic variants, or known disease risk factors.

Panel	C9orf72 (inc/ex)	Cohort (n)	Number of monogenic or RF carriers	Oligogenic cases (>1 variant)	Number of carriers within oligogenic cases	Fisher's test (p-value)
**>1 variant: full FTD-ALS panel (MAF 5%)**	inc	244	33	48	**17**	0.0001
**>1 variant: full FTD-ALS panel (MAF 5%)**	ex	244	33	43	**12**	0.0054
**>1 variant: full FTD-ALS panel (MAF 1%)**	inc	244	33	19	**11**	0.0001
**>1 variant: full FTD-ALS panel (MAF 1%)**	ex	244	33	15	**7**	0.0013
**>1 variant: medium FTD-ALS panel (MAF 5%)**	inc	244	33	15	**9**	0.0001
**>1 variant: medium FTD-ALS panel (MAF 5%)**	ex	244	33	11	4	0.0461
**>1 variant: medium FTD-ALS panel (MAF 1%)**	inc	244	33	7	5	0.0006
**>1 variant: medium FTD-ALS panel (MAF 1%)**	ex	244	33	4	2	0.0895
**>1 variant: AD panel (MAF 5%)**		277	36	8	7	0.0001
**>1 variant: AD panel (MAF 1%)**		277	36	6	6	0.0001
**>1 variant: PD-DLB panel (MAF 5%)**		97	16	39	12	0.004
**>1 variant: PD-DLB panel (MAF 1%)**		97	16	23	10	0.0003

Supplementary table 4. A table highlighting the enrichment of cases harbouring established monogenic or risk factor cases within cases that harbour >1 variant.

	>1 variant: full FTD-ALS panel (5% MAF)		>1 variant: full FTD-ALS panel (1% MAF)		>1 variant: medium FTD-ALS panel (5% MAF)		>1 variant: medium FTD-ALS panel (1% MAF)		>1 variant in AD panel (5% MAF)	>1 variant in AD panel (1% MAF)	>1 variant in PD-DLB panel (5% MAF)	>1 variant in PD-DLB panel (1% MAF)
	**inc *C9orf72***	**ex *C9orf72***	**inc *C9orf72***	**ex *C9orf72***	**inc *C9orf72***	**ex *C9orf72***	**inc *C9orf72***	**ex *C9orf72***	**N/A**			
**Sensitivity (%)**	51.50	36.36	33.33	21.21	27.27	12.12	15.15	6.06	19.44	16.67	75.00	62.50
**95% Sensitivity CI**	33.50-69.20	20.40-54.88	17.96-51.83	8.98-38.91	13.30-45.52	3.40-28.20	5.11-31.90	0.74-20.23	8.19-36.02	6.37-32.81	47.62-92.73	35.43-84.80
**Specificity (%)**	85.30	86.64	96.21	96.21	97.16	96.68	99.05	99.05	99.59	100.00	66.67	83.95
**95% specificity CI**	79.80-89.80	81.57-90.74	92.67-98.35	92.67-98.35	93.91-98.95	93.28-98.66	96.62-99.89	96.62-99.89	97.71-99.99	98.48-100	55.32-76.76	74.12-91.17
**PLR**	3.50	2.72	8.79	5.59	9.59	3.65	15.98	6.39	46.86		2.25	3.89
**95% PLR CI**	2.2-5.6	1.56-4.75	3.82-20.23	2.17-14.40	3.65-25.19	1.13-11.80	3.23-79.04	0.93-43.85	5.94-369.83		1.48-3.42	2.08-7.28
**NLR**	0.57	0.73	0.69	0.82	0.75	0.91	0.86	0.95	0.81	0.83	0.38	0.45
**95% NLR CI**	0.40-0.81	0.56-0.96	0.54-0.88	0.68-0.98	0.61-0.92	0.80-1.03	0.74-0.99	0.87-1.04	0.69-0.95	0.72-0.96	0.16-0.89	0.24-0.87
**PPV (%)**	35.42	27.91	57.89	46.67	60.00	36.36	71.34	50.00	87.50	100.00	30.77	43.48
**95% PPV CI**	22.16-50.54	15.33-43.67	33.50-79.75	21.27-73.41	32.39-83.66	10.93-69.21	29.04-96.33	6.76-93.24	47.35-99.68	54.07-100	17.02-47.57	23.19-65.51
**NPV (%)**	91.84	90.54	90.22	88.65	89.52	87.55	88.19	87.08	89.22	88.93	93.10	91.89
**95% NPV CI**	87.08-95.26	85.90-94.05	85.57-93.77	83.81-92.45	84.81-93.17	82.62-91.50	83.38-92.00	82.17-91.05	84.89-92.66	84.57-92.41	83.27-98.09	83.18-96.67

Supplementary table 5. The sensitivity, specificity, Positive Likelihood Ratio (PLR), Negative Likelihood Ratio (NLR), Positive Predicative Value (PPV), and Negative Predicative Value (NPV) that an affected individual would have a highly penetrant allele, or risk factor for their disease upon the observation of >1 variant in the relevant panel at the relevant Minor Allele Frequency (MAF%) as indicated. These data are illustrated in Figure 1.

	Controls (n=362)	Controls with ≥1 variant. N (%)	Total number of monogenic cases	Monogenic cases with an extra non-pathogenic variant	Percentage of monogenic cases with a non-pathogenic variant	Fisher's test (p-value)
**>1 variant: full FTD-ALS panel (MAF 5%) inc C9orf72**	362	174 (48.07)	33	12	36.36	0.21
**>1 variant: full FTD-ALS panel (MAF 5%)**	362	174 (48.07)	19	9	47.37	0.12
**>1 variant: full FTD-ALS panel (MAF 1%) inc C9orf72**	362	117 (32.32)	33	9	27.27	0.68
**>1 variant: full FTD-ALS genes (MAF 1%)**	362	117 (32.32)	19	7	36.84	0.80
**> 1 variant: AD panel (MAF 5%)**	362	78 (21.55)	36	7	19.44	1.00
**> 1 variant: AD panel (MAF 1%)**	362	50 (13.81)	36	6	16.67	0.62
**> 1 variant: PD-DLB genes (MAF 5%)**	362	229 (63.26)	16	12	75.00	0.43
**> 1 variant: PD-DLB genes (MAF 1%)**	362	158 (43.64)	16	10	62.50	0.20

Supplementary table 6. Number of controls with any variant across the relevant disease panel at the relevant threshold. A Fisher's test was performed to see if cases containing a known monogenic or risk factor variant within that panel were more likely that controls to also have an additional non-pathogenic variant.

	Age of onset (years)			Age of death (years)			Disease duration (years)		
	**>1 variant**	**≤1 variant**	**p-value**	**>1 variant**	**≤1 variant**	**p-value**	**>1 variant**	**≤1 variant**	**p-value**
**FTD-ALS (MAF 5%) (mean, SD)**	55.40 (11.60)	61.2 (12.10)	0.17	65.5 (13.30)	65.32 (11.60)	0.94	10.70 (10.80)	5.90 (4.30)	0.020
**FTD-ALS (MAF 1%) (mean, SD)**	56.00 (16.70)	60.5 (12.00)	0.54	73 (11.70)	65.04 (11.80)	0.062	18.00 (15.60)	6.30 (4.80)	0.00060
**PD-DLB (MAF 5%) (mean, SD)**	62.97 (11.99)	64.18 (9.26)	0.989	73.77 (10.49)	75.05 (7.29)	0.54	7.87 (4.22)	9.62 (5.73)	0.282
**PD-DLB (MAF 1%) (mean, SD)**	66.1 (9.92)	63.30 (10.22)	0.432	75.54 (7.18)	74.64 (7.75)	0.97	7.56 (4.30)	9.32 (5.49)	0.432

Supplementary table 7. Mean (SD) age of disease onset, death and duration for all cases in their relevant cohort after the removal of individuals with known highly penetrant alleles or disease risk factors. A longer disease duration was observed in cases of FTD-ALS with >1 variant compared to those with ≤ 1 variant.

Case	Age onset	Age death	Disease duration	FH_of_disease	Monogenic?	Chromosome	Position	Reference Allele	Sample Allele	Variation Type	Gene Symbol	Protein Variant	Variant interpretation	Translation Impact	SIFT Function Prediction	SIFT Score	PolyPhen-2 Function Prediction	CADD Score	Conservation phyloP p-value	ExAC Frequency
**1**		71		N	Y	7	94944735	A	G	SNV	PON1	p.L90P	UC	missense	D	0	PrD	29.6	0.00003258	0.028
						17	42430146	G	T	SNV	GRN	p.A588S	LB	missense	D	0.04	B	13.92		0.046
						9	*C9orf72*						P							
**2**		74		N	N	7	94928347	G	C	SNV	PON1	p.T326R	UC	missense	T	1	B	10.21		0.001
						14	21161931	A	G	SNV	ANG	p.I70V	LB	missense	T	0.3	B			0.061
**3**		83		N	N	5	138665061	C	T	SNV	MATR3	p.R553C;	LB	missense			B	23.1		0.011
						12	49689404	G	T	SNV	PRPH	p.D141Y	UC	missense	D	0.01	PoD	27.4	0.0004159	0.247
**4**		87		N	N	2	74598723	T	C	SNV	DCTN1	p.I159V;	UC	missense	T	1	B		0.005445	0.799
						9	35059655		T	Ins	VCP	p.N616fs*12	LB	frameshift						
						17	42429414	G	T	SNV	GRN	p.C404F	LB	missense	D	0	PrD	32	0.000005176	
						17	44067341	C	T	SNV	MAPT	p.S427F	B	missense	D	0.05	PrD	28.5	0.001259	0.146
**5**	73	75	2	N	Y	9	135224754	T	C	SNV	SETX	p.Y21C	UC	missense	D	0	PrD	24	0.0004831	0.001
						9	*C9orf72*						P							
**6**	41	77	36	N	N	9	35059655		T	Ins	VCP	p.N616fs*12	LB	frameshift						
						20	57016044	TCT		Del	VAPB	p.S160del	LB	in-frame						
**7**	52	56	4	N	Y	7	94953733	T	C	SNV	PON1	p.N19D	UC	missense	T	0.51	B			0.159
						9	*C9orf72*						P							
**8**	55	60	5	Y	Y	7	95024007	G	A	SNV	PON3	p.R32*	UC	stop gain				36		0.142
						17	42426621		CCTG	Ins	GRN	p.C31fs*35	P	frameshift					0.00134	

**9**	57	60	3	N	Y	20	57016044	TCT		Del	VAPB	p.S160del	LB	in-frame						
						9	*C9orf72*						P							
**10**	53	63	10	N	0	5	179250971	C	T	SNV	SQSTM1	p.R55C;	US	missense	D	0	PrD	32	0.006209	0.003
						7	94937419	G	A	SNV	PON1	p.A201V	UC	missense	T	0.47	B	23.3		0.158
**11**	49	50	1	Y	Y	9	135140020	A	G	SNV	SETX	p.I2547T	UC	missense	T	0.66	B			0.342
						9	*C9orf72*						P							
**12**	74	82	8	N	N	9	34635620	C	A	SNV	SIGMAR1	p.A155S	UC	missense				22.7		
						9	135140020	A	G	SNV	SETX	p.I2547T	UC	missense	T	0.66	B			0.342
**13**		71		Y	Y	2	202625862	C	A	SNV	ALS2	p.R285S	UC	missense			B			
						21	33039672	T	C	SNV	SOD1	p.I114T	P	missense	D	0.01	PrD	26.3	0.00003334	
**14**		55		Y	Y	5	179252184	A	G	SNV	SQSTM1	p.K154E	LB	missense	T	0.11	B	24.4		0.242
						9	135224757	C	T	SNV	SETX	p.R20H	UC	missense	T	0.15	B			0.906
						9	*C9orf72*						P							
**15**		62		N	Y	7	95024007	G	A	SNV	PON3	p.R32*	UC	stop gain				36		0.142
						9	135224757	C	T	SNV	SETX	p.R20H	UC	missense	T	0.15	B			0.906
**16**		78		N	Y	12	54677634	G	C	SNV	HNRNPA1	p.G316R	LP	missense	D	0.02	PrD	23.3	0.000008091	
						2	74592252	C	T	SNV	DCTN1	p.R1042Q;	LB	missense	T	0.31	PoD	26.3	0.000001679	0.099
**17**		53		N	N	21	33039672	T	C	SNV	SOD1	p.I114T	P	missense	D	0.01	PrD	26.3	0.00003334	
						9	34635679	G	A	SNV	SIGMAR1	p.R188W;	UC	missense	D	0.01	PrD	34		0.778
**18**		65		Y	N	9	135202325	A	C	SNV	SETX	p.C1554G	UC	missense	D	0.03	PrD	21.6	0.000526	0.584
						7	94953733	T	C	SNV	PON1	p.N19D	UC	missense	T	0.51	B			0.159
**19**		73		N	Y	17	42430146	G	T	SNV	GRN	p.A588S	LB	missense	D	0.04	B	13.92		0.046

						12	49689404	G	T	SNV	PRPH	p.D141Y	P	missense	D	0.01	PoD	27.4	0.0004159	0.247

Supplementary table 8. All variants in FTD-ALS cases with >1 variant in the full gene panel (28 genes) at a threshold of 1% MAF. The age of onset, death and disease duration is shown where available, together with all variant data. Key – ACMG – American College of Medical Genetics, B- Benign, LB – Likely Benign, UC – Unclassified, US – Uncertain Significance, RF – Risk Factor, LP – Likely Pathogenic, P – Pathogenic, T – Tolerated, D - Deleterious.

Case	Sub-phenotype	Age onset	Age death	Disease duration	FH of disease	McKeith Criteria	Monogenic ?	APOE genotype	Chromosome	Position	Reference Allele	Sample Allele	Variation Type	Gene Symbol	Protein Variant	ACMG Variant interpretation	Translation Impact	SIFT Function Prediction	SIFT Score	PolyPhen-2 Function Prediction	CADD Score	Conservation phyloP p-value	ExAC Frequency
1	DLB		79			Neo		3/3	3	184046450	A	G	SNV	EIF4G1	p.M1336V	LB	missense	D	0.01	PoD	26.3	0.00001702	0.022
									17	42430146	G	T	SNV	GRN	p.A588S	LB	missense	D	0.04	B	13.92		0.046
2	DLB		81			Limbic		3/4	15	44865000	T	C	SNV	SPG11	p.N2075S	UC	missense	T	0.65	B			0.259
									17	42428756	G	C	SNV	GRN	p.E287D	LB	missense	T	0.32	PoD	23.9		0.003
3	DLB	86	88	2		Neo		3/4	2	74759825	G	A	SNV	HTRA2	p.G399S	UC	missense	D	0.02	PrD	24.1	0.00052	0.437
									3	184044687	C	T	SNV	EIF4G1	p.P1075L	US	missense	T	0.14	B	26.6	0.000003873	
4	PD		80					3/3	3	184046529	T	C	SNV	EIF4G1	p.M1355T	US	missense	D	0	PrD	26.8	0.0005649	0.04
									15	44949354	C	T	SNV	SPG11	p.V270I	UC	missense	T	0.09	PoD	23.6	0.0001435	0.609
5	DLB	73	78	5				4/4	2	233712223	T	A	SNV	GIGYF2	p.L1230Q	LB	missense	T	0.4	B			0.002
									15	44907562	T	C	SNV	SPG11	p.K1013E	UC	missense	T	0.21	B	15.76		0.993
6	DLB	75	77	2				3/3	2	233712223	T	A	SNV	GIGYF2	p.L1230Q	LB	missense	T	0.4	B			0.002
									6	41129207	C	T	SNV	TREM2	p.R62H	RF	missense			B	11.11		0.826
7	PD	60	75	15		Neo		3/4	1	227073271	C	T	SNV	PSEN2	p.S130L	LB	missense	D	0.02	PoD	31	0.00006714	0.064
									3	184041256	G	C	SNV	EIF4G1	p.A717P	LB	missense	T	0.3	B	15.94		0.074
									6	41129207	C	T	SNV	TREM2	p.R62H	RF	missense			B	11.11		0.826
8	PD	68	77	9		Neo		3/4	1	155206167	C	T	SNV	GBA	p.E278K	RF	missense	T	0.88	B	17.33		0.979
									6	162683724	G	T	SNV	PARK2	p.A82E	UC	missense	T	1	B			0.472
									19	45411110	T	C	SNV	APOE	p.L46P	RF	missense	T	0.07	PoD	11.43		0.242
9	DLB	57	69	12		Limbic		3/3	12	40713856	G	C	SNV	LRRK2	p.E1632Q	UC	missense			PrD	24.3	0.000002056	
									22	32894483	GTCG		Del	FBXO7	p.R399fs	UC	frameshift					0.00003048	
10	DLB	65	70	5				3/3	6	41129207	C	T	SNV	TREM2	p.R62H	RF	missense			B	11.11		0.826
									6	161771219	G	A	SNV	PARK2	p.P409L	PR	missense	D	0.01	PrD	27.7	0.0001127	0.15
									12	40713899	T	C	SNV	LRRK2	p.M1646T	RF	missense			B	17.91	0.00001683	0.916
11	DLB	52	68	16		Neo		3/4	3	184043401	G	A	SNV	EIF4G1	p.R1039Q	US	missense	T	0.24	B	24.1	0.001589	
									12	40713899	T	C	SNV	LRRK2	p.M1646T	RF	missense			B	17.91	0.00001683	0.916
12	DLB	62	72	10		Neo		3/3	1	20960395	C	A	SNV	PINK1	p.S118R	UC	missense	T	0.32	B	16.26		
									1	155206037	G	A	SNV	GBA	p.T321M	RF	missense	T	0.11	B	22.2		0.657

									2	233712223	T	C	SNV	GIGYF2	p.L1230P	LB	missense	T	0.23	B			0.021
									15	44949354	C	T	SNV	SPG11	p.V270I	UC	missense	T	0.09	PoD	23.6	0.0001435	0.609
13	PD	58	63	5		Limbic		3/4	6	41129252	C	T	SNV	TREM2	p.R47H	RF	missense			PrD	33		0.206
									12	40745375	G	T	SNV	LRRK2	p.C2139F	UC	missense			PrD	33	0.000004074	
14	DLB	62	75	13		Limbic		3/3	1	155206167	C	T	SNV	GBA	p.E278K	RF	missense	T	0.88	B	17.33		0.979
									17	42429839	G	C	SNV	GRN	p.G515A	UC	missense	T	0.56	B			0.268
15	PD		76			Bstem		3/4	6	161771219	G	A	SNV	PARK2	p.P409L	PR	missense	D	0.01	PrD	27.7	0.0001127	0.15
									12	40713899	T	C	SNV	LRRK2	p.M1646T	RF	missense			B	17.91	0.00001683	0.916
16	DLB	65	66	1				3/4	1	155205043	A	G	SNV	GBA	p.L434P	RF	missense	D	0.04	PoD	24.8	0.0002443	0.31
									17	42426585	C	T	SNV	GRN	p.T18M	LB	missense	T	0.08	PoD	25.8		0.003
17	PD	66	69	3		Limbic		2/3	1	155205043	A	G	SNV	GBA	p.L434P	RF	missense	D	0.04	PoD	24.8	0.0002443	0.31
									12	40677726	G	T	SNV	LRRK2	p.S764I	UC	missense			B	16.12		
18	PD	63	70	7		Limbic		3/4	6	162206852	G	A	SNV	PARK2	p.R275W	PR	missense	D	0	PrD	34	0.006412	0.206
									19	45412358	C	G	SNV	APOE	p.R269G	UC	missense	D	0.01	B	25.6		0.042
19	PD	40	69	29		Limbic		3/4	6	41129100	G	A	SNV	TREM2	p.R98W	RF	missense			PoD	25.2		0.007
									6	161771240	C	T	SNV	PARK2	p.G430D	PR	missense	D	0	PrD	33	0.0001127	0.011
									6	162206852	G	A	SNV	PARK2	p.R275W	PR	missense	D	0	PrD	34	0.006412	0.206
20	PD	54	64	10		Neo		3/4	2	233712223	T	C	SNV	GIGYF2	p.L1230P	LB	missense	T	0.23	B			0.021
									12	40713899	T	C	SNV	LRRK2	p.M1646T	RF	missense			B	17.91	0.00001683	0.916
21	DLB		94			Neo		3/4	1	227076537	C	A	SNV	PSEN2	p.L192I	US	missense	T	0.15	B	25.4		
									6	41129207	C	T	SNV	TREM2	p.R62H	RF	missense			B	11.11		0.826
22	DLB		80			Neo		3/3	3	184039828	C	T	SNV	EIF4G1	p.P399S	LB	missense	T	1	B			0.079
									6	41129207	C	T	SNV	TREM2	p.R62H	RF	missense			B	11.11		0.826
23	DLB		74			Neo		3/3	1	155205043	A	G	SNV	GBA	p.L434P	RF	missense	D	0.04	PoD	24.8	0.0002443	0.31
									2	74759825	G	A	SNV	HTRA2	p.G399S	UC	missense	D	0.02	PrD	24.1	0.00052	0.437

Supplementary table 9. Variants in PD-DLB cases with >1 variant in the full gene panel (28 genes) at a threshold of 1% MAF. The age of onset, death and disease duration is shown where available, together with all variant data. Key – ACMG – American College of Medical Genetics, B- Benign, LB – Likely Benign, UC – Unclassified, US – Uncertain Significance, RF – Risk Factor, PR – Pathogenic Recessive, LP – Likely Pathogenic, P – Pathogenic, T – Tolerated, D - Deleterious. Cases highlighted in blue type indicate those individuals with >1 pathogenic, likely pathogenic or known risk factor for a neurodegenerative disease based on ACMG criteria.

REFERENCES

1. Braak H, Alafuzoff I, Arzberger T, et al. Staging of Alzheimer disease-associated neurofibrillary pathology using paraffin sections and immunocytochemistry. Acta neuropathologica 2006;**112**(4):389-404 doi: 10.1007/s00401-006-0127-z[published Online First: Epub Date]|.
2. Keogh MJ, Wei W, Wilson I, et al. Genetic compendium of 1511 human brains available through the UK Medical Research Council Brain Banks Network Resource. Genome Res 2017;**27**(1):165-73 doi: 10.1101/gr.210609.116[published Online First: Epub Date]|.
3. Langmead B, Trapnell C, Pop M, et al. Ultrafast and memory-efficient alignment of short DNA sequences to the human genome. Genome Biol 2009;**10**(3):R25 doi: 10.1186/gb-2009-10-3-r25[published Online First: Epub Date]|.
4. Garrison EM, G. Haplotype-based variant detection from shortread sequencing. arXiv 2012:1207.3907v2
5. Genomes Project C, Abecasis GR, Auton A, et al. An integrated map of genetic variation from 1,092 human genomes. Nature 2012;**491**(7422):56-65 doi: 10.1038/nature11632[published Online First: Epub Date]|.
6. Exome_Variant_Server. NHLBI GO Exome Sequencing Project (ESP). Secondary NHLBI GO Exome Sequencing Project (ESP) 2016. http://evs.gs.washington.edu/EVS/.
7. NHLBI GO Exome Sequencing Project (ESP). Secondary NHLBI GO Exome Sequencing Project (ESP). URL: http://evs.gs.washington.edu/EVS/.
8. Lek M, Karczewski KJ, Minikel EV, et al. Analysis of protein-coding genetic variation in 60,706 humans. Nature 2016;**536**(7616):285-91 doi: 10.1038/nature19057[published Online First: Epub Date]|.
9. Exome Aggregation Consortium (ExAC). Secondary Exome Aggregation Consortium (ExAC). http://exac.broadinstitute.org.
10. Richards S, Aziz N, Bale S, et al. Standards and guidelines for the interpretation of sequence variants: a joint consensus recommendation of the American College of Medical Genetics and Genomics and the Association for Molecular Pathology. Genetics in medicine : official journal of the American College of Medical Genetics 2015;**17**(5):405-24 doi: 10.1038/gim.2015.30[published Online First: Epub Date]|.
11. MacArthur DG, Manolio TA, Dimmock DP, et al. Guidelines for investigating causality of sequence variants in human disease. Nature 2014;**508**(7497):469-76 doi: 10.1038/nature13127[published Online First: Epub Date]|.
12. Cady J, Allred P, Bali T, et al. Amyotrophic lateral sclerosis onset is influenced by the burden of rare variants in known amyotrophic lateral sclerosis genes. Annals of neurology 2015;**77**(1):100-13 doi: 10.1002/ana.24306[published Online First: Epub Date]|.
13. Renton AE, Majounie E, Waite A, et al. A hexanucleotide repeat expansion in C9ORF72 is the cause of chromosome 9p21-linked ALS-FTD. Neuron 2011;**72**(2):257-68 doi: 10.1016/j.neuron.2011.09.010[published Online First: Epub Date]|.
14. Conte A, Lattante S, Zollino M, et al. P525L FUS mutation is consistently associated with a severe form of juvenile amyotrophic lateral sclerosis.

Neuromuscular disorders : NMD 2012;**22**(1):73-5 doi: 10.1016/j.nmd.2011.08.003[published Online First: Epub Date]|.
15. Leblond CS, Webber A, Gan-Or Z, et al. De novo FUS P525L mutation in Juvenile amyotrophic lateral sclerosis with dysphonia and diplopia. Neurology. Genetics 2016;**2**(2):e63 doi: 10.1212/NXG.0000000000000063[published Online First: Epub Date]|.
16. Rosen DR, Siddique T, Patterson D, et al. Mutations in Cu/Zn superoxide dismutase gene are associated with familial amyotrophic lateral sclerosis. Nature 1993;**362**(6415):59-62 doi: 10.1038/362059a0[published Online First: Epub Date]|.
