# Supplementary material for: Oligogenic genetic variation of neurodegenerative disease genes in 980 postmortem human brains
Source: J Neurol Neurosurg Psychiatry. 2018 Jan 13;89(8):813–6. doi: 10.1136/jnnp-2017-317234 (PMC6204946; doi:10.1136/jnnp-2017-317234)
Supplement: Supplementary file 2 [file jnnp-2017-317234supp002.pdf]

Modelling Oligovariants and Monogenicity with the Categorical Distribution

1. Model

Let us assume that we are presented with a case patient, with a state $X \in \{1, 2, 3, 4\}$ where

$$X = 1 \implies \text{“} > 1 \text{ variant and monogenic”} \tag{1}$$
$$X = 2 \implies \text{“} > 1 \text{ variant and not monogenic”} \tag{2}$$
$$X = 3 \implies \text{“} \leq 1 \text{ variant and monogenic”} \tag{3}$$
$$X = 4 \implies \text{“} \leq 1 \text{ variant and not monogenic”}. \tag{4}$$

We can model this as each patient being a roll of a 4-sided die with outcome X. Note that X here is a random variable. Given N observed patients, we wish to make predictions about the probability that a new patient will be in one of the above categories. To do this, we take a Bayesian approach as outlined in [1].

2. Likelihood

We begin by writing down the likelihood for a categorical distribution, which is a model for a K-sided die (a higher-dimensional analogue of the Bernoulli distribution for a coin flip)

$$\mathbb{P}(\mathcal{D}|\boldsymbol{\theta}) = \prod_{k=1}^{4} \theta_k^{N_k} \tag{5}$$

where θ_k is the probability of outcome k (where $k \in \{1, \dots, 4\}$), N_k is the number of patients observed with outcome k and $\mathcal{D} = \{N_1, \dots, N_4\}$ is the data. $\boldsymbol{\theta}$ is the set of all parameters θ_k. Hence the probability of observing the entire dataset is simply the product of probabilities for each individual outcome.

3. Priors

In order to access the posterior, which is the quantity of interest here ($\mathbb{P}(\boldsymbol{\theta}|\mathcal{D})$), we require a prior $\mathbb{P}(\boldsymbol{\theta})$ which encodes our belief about the parameters we wish to infer ($\boldsymbol{\theta}$) before we see any data.

For mathematical convenience, we choose the prior to be a Dirichlet distribution

$$\mathrm{Dir}(\boldsymbol{\theta}|\boldsymbol{\alpha}) = \frac{1}{B(\alpha)} \prod_{k=1}^{K} \theta_k^{\alpha_k - 1} \mathbb{I}(\boldsymbol{\theta} \in S_K) \quad (6)$$

$$S_K = \{\boldsymbol{\theta} : 0 \leq \theta_k \leq 1, \sum_{k=1}^{K} \theta_k = 1\} \quad (7)$$

$$B(\boldsymbol{\alpha}) \equiv \frac{\prod_{k=1}^{K} \Gamma(\alpha_k)}{\Gamma(\alpha_0)} \quad (8)$$

$$\alpha_0 \equiv \sum_{k=1}^{K} \alpha_k \quad (9)$$

where $\Gamma(x)$ is the gamma-function and $\mathbb{I}(z)$ is the indicator function. Although the Dirichlet distribution appears to be complex, it is a natural class of distribution to describe the probabilities of a weighted die. The reason for this is that the distribution is constrained such that the sum of probabilites of each possible outcome (θ_k) is exactly 1 (see Eq.(7)). The shape of the Dirichlet distribution may be tuned to encode our belief on $\boldsymbol{\theta}$ by appropriately choosing $\boldsymbol{\alpha}$. We choose $\boldsymbol{\alpha} = (1, 1, 1, 1)$ which is a multidimensional uniform prior on the space S_k (this is called an "uninformative" prior).

4. Posterior

Our choice of prior is convenient because it is a *conjugate* prior. In other words, the data simply changes the parameters of the prior, whilst retaining its family (namely the Dirichlet distribution). It can be shown [1] that

$$\mathbb{P}(\boldsymbol{\theta}|\mathcal{D}) = \mathrm{Dir}(\boldsymbol{\theta}|\alpha_1 + N_1, \ldots, \alpha_K + N_K). \quad (10)$$

Given the data $\mathcal{D} = \{N_1 = 7, N_2 = 1, N_3 = 29, N_4 = 240\}$, and our prior, the posterior distribution is therefore

$$\mathbb{P}(\boldsymbol{\theta}|\mathcal{D}) = \mathrm{Dir}(\boldsymbol{\theta}|\alpha_1 = 8, \alpha_2 = 2, \alpha_3 = 30, \alpha_4 = 241). \quad (11)$$

This distribution encodes all of the uncertainty given the data. We may use this to make predictions about future patients.

5. Posterior predictive

5.1. Classifying a patient in any category

We may be interested in the probability that a future patient falls into one of the above categories, i.e. $\mathbb{P}(X = j|\mathcal{D})$. To do this, we wish to integrate over our parametric uncertainty in $\boldsymbol{\theta}$. We can do this by using the posterior distribution

$$\mathbb{P}(X = j|\mathcal{D}) = \int \mathbb{P}(X = j|\boldsymbol{\theta})\mathbb{P}(\boldsymbol{\theta}|\mathcal{D})d\boldsymbol{\theta} \quad (12)$$

where $\mathbb{P}(X = j|\boldsymbol{\theta})$ is simply θ_j. It turns out that this takes a particularly intuitive form

$$\mathbb{P}(X = j|\mathcal{D}) = \frac{\alpha_j + N_j}{\alpha_0 + N}. \quad (13)$$

In other words, the probability that a future patient will be given a particular classification is the fraction of times a patient has already been given that classification, with some correction terms from the prior. These correction terms allow for the possibility of $N_j = 0$ and still give $\mathbb{P}(X = j|\mathcal{D}) > 0$,

hence solving the zero-count problem. Note that for small amounts of data the prior has a more dominant role in the posterior predictive probability. For $j = 1$, we have

$$\mathbb{P}(X = 1|\mathcal{D}) = \frac{1+7}{4+277} = 0.03\% \text{ (1 s.f.)} \tag{14}$$

5.2. Predicting monogenecity given oligovariants

In this case, we are interested in a slightly different object, namely the probability that $X = 1$ (monogenic and has >1 variants) given that $X = 1$ or 2 (has > 1 variants) and the data. We can solve for this using Bayes rule

$$\mathbb{P}(X = 1|X \in \{1,2\}, \mathcal{D}) = \frac{\mathbb{P}(X \in \{1,2\}|X = 1, \mathcal{D})\mathbb{P}(X = 1|\mathcal{D})}{\mathbb{P}(X \in \{1,2\}|D)} \tag{15}$$

but $\mathbb{P}(X \in \{1,2\}|X = 1, \mathcal{D}) = 1$ and $\mathbb{P}(X \in \{1,2\}|D) = \mathbb{P}(X = 1|\mathcal{D}) + \mathbb{P}(X = 2|\mathcal{D})$ since the events are disjoint (a patient cannot be classified as both $X = 1$ and $X = 2$). Hence, by using Eq.(13), we find that

$$\mathbb{P}(X = 1|X \in \{1,2\}, \mathcal{D}) = \frac{\alpha_1 + N_1}{\alpha_1 + \alpha_2 + N_1 + N_2} \tag{16}$$

which is again intuitive in its form. In our case

$$\mathbb{P}(X = 1|X \in \{1,2\}, \mathcal{D}) = \frac{1+7}{1+1+7+1} = 80\%. \tag{17}$$

Therefore the Bayesian posterior predictive probability that a patient showing multiple variants is monogenic is 80%, given a uniform prior distribution.

References

[1] **Murphy KP**. 2012. *Machine learning: a probabilistic perspective.* MIT press.
